# Squalene Inhibits ATM-Dependent Signaling in γIR-Induced DNA Damage Response through Induction of Wip1 Phosphatase

**DOI:** 10.1371/journal.pone.0147570

**Published:** 2016-01-29

**Authors:** Naoto Tatewaki, Tetsuya Konishi, Yuki Nakajima, Miyako Nishida, Masafumi Saito, Takahiro Eitsuka, Toshiyuki Sakamaki, Nobuo Ikekawa, Hiroshi Nishida

**Affiliations:** 1 Department of Applied Life Sciences, Niigata University of Pharmacy and Applied Life Sciences, Niigata, Japan; 2 Department of Pharmaceutical Sciences, Niigata University of Pharmacy and Applied Life Sciences, Niigata, Japan; 3 Niigata Bio Research Center, Niigata, Japan; German Cancer Research Center, GERMANY

## Abstract

Ataxia telangiectasia mutated (ATM) kinase plays a crucial role as a master controller in the cellular DNA damage response. Inhibition of ATM leads to inhibition of the checkpoint signaling pathway. Hence, addition of checkpoint inhibitors to anticancer therapies may be an effective targeting strategy. A recent study reported that Wip1, a protein phosphatase, de-phosphorylates serine 1981 of ATM during the DNA damage response. Squalene has been proposed to complement anticancer therapies such as chemotherapy and radiotherapy; however, there is little mechanistic information supporting this idea. Here, we report the inhibitory effect of squalene on ATM-dependent DNA damage signals. Squalene itself did not affect cell viability and the cell cycle of A549 cells, but it enhanced the cytotoxicity of gamma-irradiation (γIR). The in vitro kinase activity of ATM was not altered by squalene. However, squalene increased Wip1 expression in cells and suppressed ATM activation in γIR-treated cells. Consistent with the potential inhibition of ATM by squalene, IR-induced phosphorylation of ATM effectors such as p53 (Ser15) and Chk1 (Ser317) was inhibited by cell treatment with squalene. Thus, squalene inhibits the ATM-dependent signaling pathway following DNA damage through intracellular induction of Wip1 expression.

## Introduction

Ataxia telangiectasia mutated (ATM) and other related protein kinases play crucial roles as master controllers in DNA damage checkpoint signaling [1–3]. When DNA damage occurs in cells, ATM phosphorylates signaling molecules such as p53, SMC1, and Chk1 to activate cell cycle checkpoints. Ataxia telangiectasia (AT) patient-derived AT cells, which lack a functional ATM, are sensitive to ionizing radiation (IR) or radiomimetic agents with DNA-modifying effects [4,5]. ATM-deficient AT cells possess defects in cellular responses to DNA double-strand breaks (DSBs) produced by IR and radiomimetic chemicals, and thus exhibit chromosomal instability and telomere shortening [1,6]. A pleiotropic phenotype characterized by cerebellar degeneration, immunodeficiency, and predisposition to cancer is frequently observed in AT patients [1].

IR including gamma ray, X-ray, and ultraviolet (UV) radiation, along with several anticancer drugs, induces an ATM-dependent DNA damage response, resulting in cell cycle arrest at the G1/S, intra-S, and G2/M checkpoints that provide time for the repair of DNA damage or for apoptosis when the extent of DNA damage is not compatible with the survival of the cell [1,2]. The DNA damage response is strictly regulated by Wip1 phosphatase through dephosphorylation of ATM to restart the cell cycle after damaged DNA is repaired [7]. Thus, DNA damage checkpoints safely prevent the carry-over of damaged DNA to the next generation of cells. In anticancer therapies, however, DNA damage control confers cancer cells with tolerance to these treatments. Therefore, modulation or inhibition of this system could enhance tumor cell death in individuals treated with chemo/radio therapies [8].

Almost all cancer cells lose p53 function [9,10] and, as a result, exhibit dysfunction of the G1/S checkpoint. The use of inhibitors of ATM itself or ATM-associated G2/M checkpoint mediators can selectively sensitize such cancer cells with defective p53 to DNA-damaging radiation and anticancer drugs [11–14]. Thus, the G2/M checkpoint could be a more useful drug target than the G1/S checkpoint in anticancer therapy. The search for specific modulators of ATM is beneficial not only to for understanding the principle functions of this kinase but also for their potential clinical application to sensitize cancer cells to anticancer therapy. Although many ATM inhibitors have been reported [11,15–17], a potent compound has yet to be discovered for targeted inhibition of the protein kinase ATM because of the lack of specificity of existing ATM inhibitors.

In the course of our search for potential ATM modulators, we found squalene, which is known to have a potential anti-tumor effect. For example, squalene was previously shown to inhibit tumor promoter activity in a mouse skin carcinogenesis model [18], and tumor progression in the same carcinogenesis model [19]. Squalene was also shown to potentiate the cytotoxicity of various anticancer agents *in vitro* [20]. However, its detailed mechanism of action remains unclear.

Here, we demonstrate that squalene modulates cellular ATM kinase through induction of Wip1 protein phosphatase.

## Materials and Methods

### Cell culture

Human adenocarcinoma A549 cells and HEK 293T cells (ATCC: American Type Culture Collection, VA, USA) were maintained in Dulbecco’s modified Eagle’s medium (DMEM) supplemented with 10% fetal bovine serum (FBS), 100 μg/mL streptomycin, and 100 units/mL penicillin. The medium and supplements were purchased from Sigma (Sigma Chemical Co., St Louis, MO, USA) and Invitrogen (CA, USA). A549 cell culture and expression assays for Flag-tagged ATM and Flag-tagged ATR in HEK293T cells were performed as described previously [21,22]. DNA damage in cells was induced by ultraviolet C irradiation (UVC; 254 nm, UVP, Inc., Upland, CA, USA) or γ-irradiation (γIR; ^137^Cs, 2 Gy/min, PS-3000SB, Pony Industry Co., Osaka, Japan).

### Squalene solution

Purified squalene was provided by Nissei Marine Industrial Co., Ltd. (Tokyo, Japan). Squalene was dissolved in ethanol at the optimum concentration and diluted 1,000-fold in culture medium. For cell treatments, the squalene/ethanol solution was further diluted in culture medium with an ethanol concentration < 0.01% v/v.

### Protein preparation

Cells were harvested by scraping in ice-cold phosphate-buffered saline (PBS). After two washes with cold PBS, proteins were extracted from the cell pellets in urea/Tris buffer (UTB; 8 mM urea, 150 mM 2-mercaptoethanol, 50 mM Tris, pH 7.5) for immunoblot analysis or immunoprecipitation (IP) buffer (10 mM Tris; 1 mM EDTA; 1 mM EGTA; 150 mM NaCl; 0.5% NP-40; 1% Triton X-100; 1 mM phenylmethanesulfonyl fluoride, PMSF; 2 μg/mL pepstatin; 2 μg/mL aprotinin; 1 mM dithiothreitol, DTT; pH 7.4) for the ATM kinase assay. Protein concentrations were determined by the Bradford assay (Bio-Rad, CA, USA).

### GC-MS analysis

A549 cells were seeded in 60-mm dishes. After incubation with various concentrations of squalene (0–300 μM) for 3 h, the cells were washed in cold PBS and harvested. For extraction of squalene, 0.8 mL of chloroform/methanol (2:1 v/v) and squalane (Wako Pure Chemical Industries, Ltd., Osaka, Japan), as an internal standard, was added to the cell pellets, which were then sonicated. After centrifugation (10,000 rpm, 5 min, 4°C), the supernatants were evaporated and dissolved in hexane for GC-MS analysis.

GC-MS analysis was performed using a Shimadzu GCMS-QP2010 SE system (Shimadzu, Kyoto, Japan). Separation was carried out on a fused silica DB-23 capillary GC column (30 m × 0.25 mm I.D., 0.25 μm film; Agilent Technologies, Inc., Santa Clara, CA, USA). Helium gas (99.999%) was used as the carrier gas at a constant flow rate of 1.45 mL/min, an injection volume of 1 μL at a split ratio of 15:0, an injector temperature of 250°C, and an ion-source temperature of 250°C. The oven temperature was programmed at 110°C with an increase of 10°C/min up to 200°C followed by 5°C/min up to 250°C, and ending with a 5 min isothermal at 250°C. The total GC running time was 24 min. Data acquisition and processing were performed using GCMSsolution Ver. 2.7 (Shimadzu, Kyoto, Japan).

### Clonogenic assay

The clonogenic assay was performed as previously described [21]. In brief, 1 h prior to DNA damage, cells were pre-incubated with squalene (0–100 μM). Cells were sham-treated or exposed to UVC or γIR. After incubation for 14 days, the cells were washed in PBS and stained with 2% (w/v) methylene blue dissolved in 50% ethanol.

### Immunoblot analysis

Immunoblot analysis was performed as described in detail previously [21,22]. Primary antibodies against ATM (Bethyl Laboratories, Montgomery, TX, USA), phospho-ATM Ser1981 (Cell Signaling Technologies, Danvers, NJ, USA), ATR (Bethyl Laboratories), phospho-ATR Ser428 (Cell Signaling Technologies), p53 (Cell Signaling Technologies), phospho-p53 Ser15 (Cell Signaling Technologies), SMC1 (Bethyl Laboratories), phospho-SMC1 Ser966 (Bethyl Laboratories), Chk1 (G-4; Santa Cruz Biotechnology, Santa Cruz, CA, USA), phospho-Chk1 Ser317 (Bethyl Laboratories), phospho-Chk1 Ser345 (Cell Signaling Technologies), Chk2 (H-300; Santa Cruz), phospho-Chk2 Thr68 (Cell Signaling Technologies), Wip1 (PPM1D; Bethyl Laboratories), Cdc25A (F-6; Santa Cruz) and GAPDH (Cell Signaling Technologies) were used for immunoblots.

### G2/M checkpoint analysis

Mitosis was evaluated as described previously [21,22]. Briefly, cells were fixed in ice-cold 70% ethanol. Permeabilization was performed by incubation in 0.25% Triton X-100 in PBS. The cell pellets were incubated with 1 μg of polyclonal rabbit phospho-histone H3 (Ser10) antibody (Upstate Biotechnology, Lake Placid, NY, USA) for specific recognition of the phosphorylated form of histone H3 at Ser10. The cells were then incubated with FITC-conjugated goat anti-rabbit IgG antibody (Sigma-Aldrich, St. Louis, MO, USA). Counter-staining with PI solution (50 μg/mL propidium iodide, 100 μg/mL RNase) was performed by incubation for 20 min at room temperature. Cells were subjected to FACS analysis on a flow cytometer (Beckman-Coulter, Brea, CA, USA).

### Kinase assay

ATM and AT and Rad3-related (ATR) kinase activities were evaluated as previously described [21,22]. Endogenous Flag-ATM and Flag-ATR were immunoprecipitated for 4 h at 4°C with 20 μg of anti-Flag^®^ M2 monoclonal antibody (Sigma-Aldrich) from 5 mg of cell lysate, followed by incubation with Protein-G Sepharose (GE Healthcare UK, Ltd.) for 1 h at 4°C. Immunocomplexes were washed twice with TGN buffer (50 mM Tris [pH 7.4], 50 mM glycerophosphate, 150 mM NaCl, 1% Tween20, and 10% glycerol) and once with kinase buffer containing 10 mM HEPES (pH 7.5), 50 mM glycerophosphate, 50 mM NaCl, 10 mM MgCl_2_, and 10 mM MnCl_2_. The phosphorylation reaction was performed by mixing 1 μg of recombinant PHAS-I (Alexis Biochemicals, CA, USA), a substrate for ATM or ATR kinase, with 10 μM ^32^P-ATP (50 Ci/mmol; MP Biomedicals, Inc., Santa Ana, CA, USA) as previously described [21–23]. After incubation for 20 min at 30°C, the reaction was terminated by adding 4× SDS sample loading buffer, and the samples were subjected to 7% SDS-PAGE. Gels were dried using a Bio-Rad Gel Dryer (Bio-Rad Laboratories) and exposed to a Storage Phosphor Screen (Amersham Biosciences, Piscataway, NJ, USA). The radioactivity incorporated in the substrate was visualized and quantified as described previously [22].

### Statistics

Statistical analysis was performed using Statistical Package for Social Science (SPSS) software. Results were considered significant when P < 0.05.

## Results

### Squalene uptake by A549 adenocarcinoma cells

**Fig 1:**
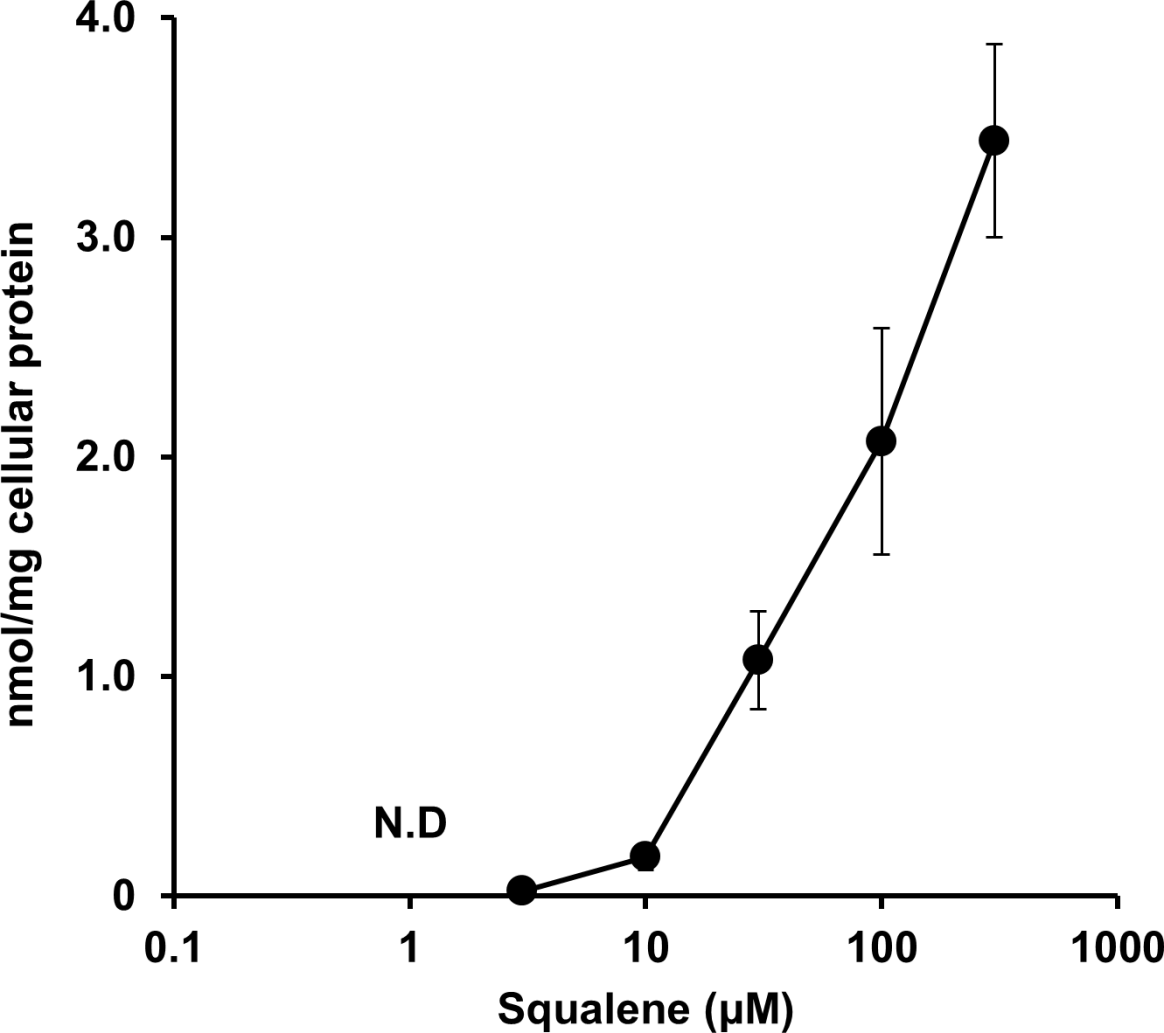
Squalene uptake in cultured cells. Squalene uptake by A549 adenocarcinoma cells. The cells were incubated with various concentrations of squalene (0–300 μM) for 3 h, washed in PBS, and harvested. Squalene was extracted from the cells using chloroform/methanol, and the amount of squalene was determined by GC-MS.

We first examined whether or not squalene is incorporated into A549 cells. The concentration of squalene in the cells increased in a concentration-dependent manner up to 300 μM (Fig 1). Endogenous squalene was not detected in A549 cells (squalene-untreated cells). Because the cells were washed in PBS repeatedly but still showed a signal, squalene incorporation in A549 adenocarcinoma cells was confirmed.

### Squalene sensitizes A549 cells to γIR toxicity

**Fig 2:**
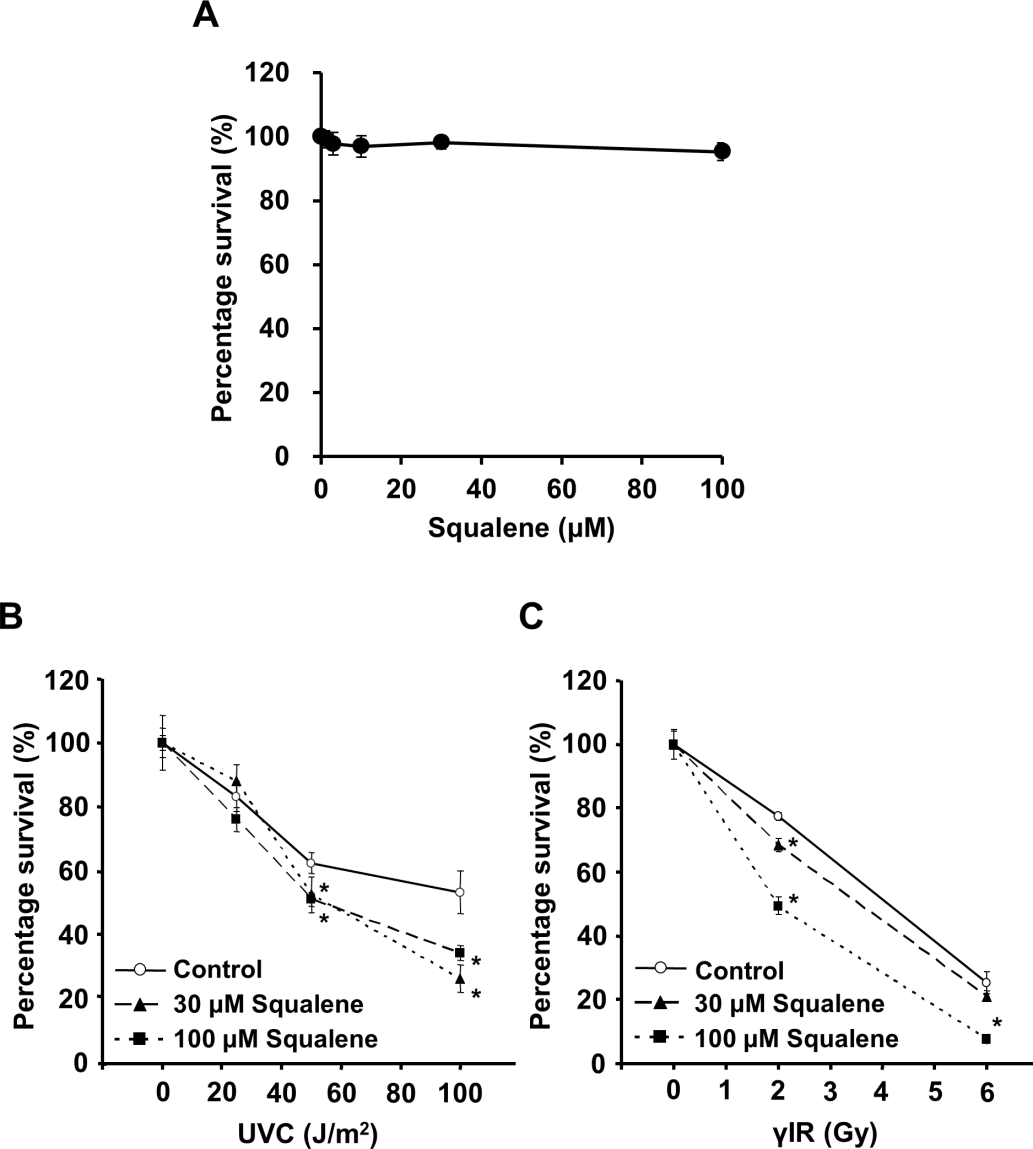
Squalene enhances γIR toxicity. Relative cell viability was measured by a clonogenic assay. (A) Cytotoxicity of squalene (0, 1, 3, 10, 30, and 100 μM) was measured after 14 days of incubation. (B) and (C) Impact of squalene on radiation toxicity. The cells were subjected to 1 h of pre-incubation and 14 days of post-incubation with squalene (0, 30, and 100 μM) after UVC irradiation (0, 25, 50, and 100 J/m^2^) or γIR (0, 2, and 6 Gy). Data are presented as the mean ± S.D. of three independent experiments (*P < 0.05 vs. untreated control).

The cytotoxicity of squalene was investigated in A549 cells. Squalene (0–100 μM) did not decrease the cell proliferation rate (Fig 2A). The effect of squalene treatment was examined on cell survival following UVC (254 nm) or γIR (^137^Cs) exposure in the presence or absence of squalene. The viability of A549 cells was determined by a clonogenic assay 14 days after UVC irradiation or γIR (Fig 2B and 2C). Squalene-untreated cells showed a concentration-dependent decrease in viability after UVC irradiation or γIR, but the cytotoxicity of radiation was significantly enhanced in squalene-treated cells exposed to over 50 J/m^2^ UVC or 2 Gy γIR. A similar result was also obtained in γIR-irradiated HEK293T cells (data not shown). These results suggest that squalene modulates the signals in the DNA damage response induced by γIR, regardless of cell type, i.e. in cells with or without mutated p53.

### Squalene inhibits the phosphorylation of checkpoint proteins

**Fig 3:**
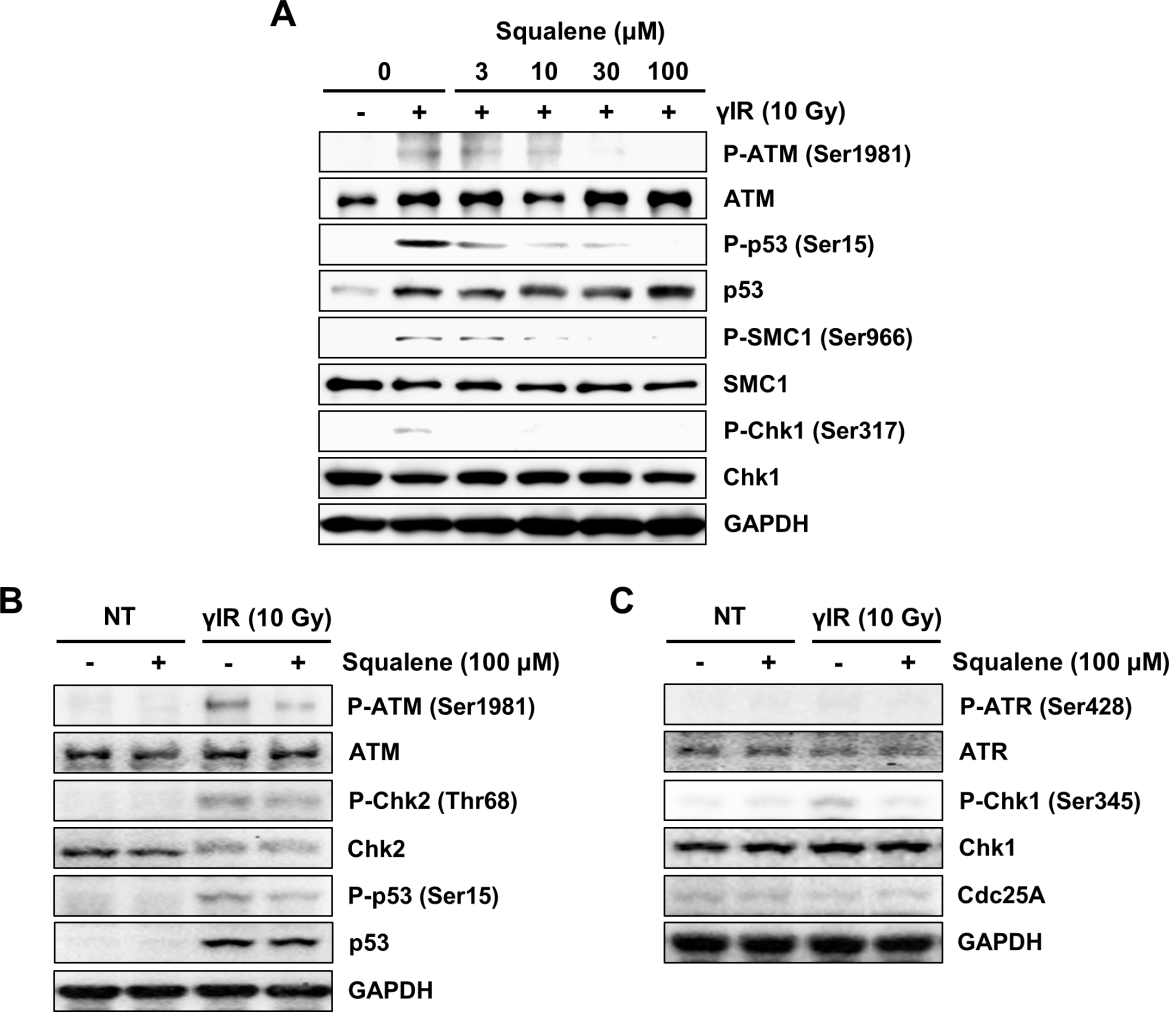
Squalene inhibits phosphorylation of checkpoint proteins after γIR. A549 adenocarcinoma cells were pre-incubated with or without squalene (3, 10, 30, and 100 μM) for 4 h followed by irradiation with γIR (10 Gy) and incubated at 37°C for 3 h. (A) Concentration-dependency of squalene on ATM, p53, SMC1, and Chk1 activation was examined by immunoblot analysis. The effect of squalene (100 μM) on the (B) ATM-Chk2-p53 pathway and (C) ATR-Chk1-Cdc25A pathway was examined by immunoblot analysis. GAPDH was used as a loading control.

To study whether or not squalene modulates DNA damage checkpoint signals activated by γIR, we examined the phosphorylation levels of p53 at serine 15 (Ser15) and those of SMC1 at serine 966 (Ser966), as these residues are direct targets of ATM [1,2,24–26], using phosphorylation site-specific antibodies. p53 phosphorylation was elevated in A549 cells irradiated with γIR (10 Gy) in the absence of squalene (Fig 3A). Moreover, p53 phosphorylation was significantly inhibited in squalene-treated cells, suggesting that squalene inhibited ATM-dependent phosphorylation of p53 at Ser15. Similarly, squalene inhibited the phosphorylation of other ATM substrates, i.e., SMC1 and Chk1, induced by γIR-generated DNA DSBs (Fig 3A). To exclude the possibility that the observed decrease in p53 and SMC1 phosphorylation was caused by squalene-induced cell cycle synchronization, the cell cycle distribution was analyzed with or without squalene treatment (data not shown). We found that squalene at 30 μM did not affect the cell cycle distribution even after 24 h of incubation. Therefore, the decreased phosphorylation of p53 and SMC1 were not caused by differential cell cycle phase distributions. Because the DNA damage checkpoint is regulated by two main pathways, the ATM-Chk2-p53 pathway and ATR-Chk1-Cdc25A pathway, it was important to determine whether squalene inhibits both of these DNA damage checkpoint pathways. Squalene inhibited the phosphorylation of ATM (Ser1981), Chk2 (Thr68), and p53 (Ser15) induced by γIR (Fig 3B). Squalene inhibited Chk1 phosphorylation (Fig 3C). However, activation of ATR and degradation of cdc25A were not significantly elevated in A549 cells irradiated with γIR (Fig 3C). Taken together, these results suggest that the suppression of γIR-induced phosphorylation of Chk2 at Thr68, p53 at Ser15, and SMC1 at Ser966 by squalene was a consequence of ATM inhibition.

### Squalene abrogates the γIR-activated G2/M checkpoint

**Fig 4:**
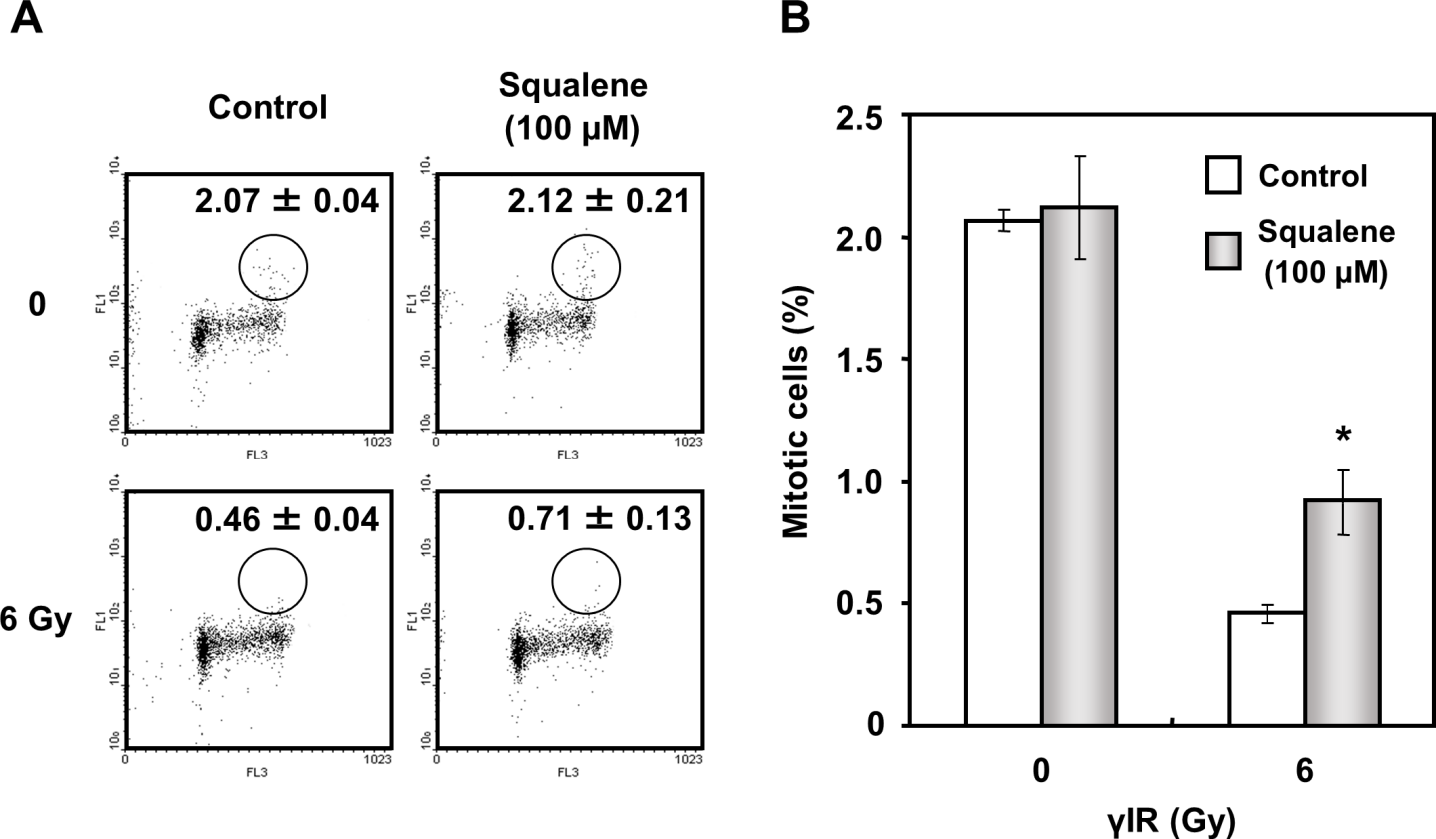
Effect of squalene on G2/M checkpoint activity. G2/M checkpoint analysis was performed by flow cytometry. (A) The percentage of mitotic cells was estimated according to the phosphorylation of histone H3 in Ser10-positive cells. A549 adenocarcinoma cells were counterstained with PI for visualization of DNA content. The cells were treated with 100 μM squalene for 4 h before and 1 h after γIR (0 and 6 Gy). (B) Data are expressed as the percentage of mitotic cells in the total number of cells. Data are presented as the mean ± S.D. of three independent experiments (*P < 0.05 vs. untreated control).

The G2/M checkpoint is a critical target for sensitizing anticancer modalities because its dysfunction promotes mitotic death in cells with extended DNA damage [27]. We examined the effect of squalene on the G2/M checkpoint function by flow cytometry. In squalene-untreated cells (control), the progression from the G2 to M phase was significantly inhibited by γIR at 6 Gy, as evidenced by a diminished mitotic cell population. However, in squalene-treated cells, the mitotic cell population recovered to the control level, indicating that squalene abolished the G2/M checkpoint activated by γIR (Fig 4). Squalene itself caused no significant change in the cell population in the G2/M phase. These results suggest that squalene modulates the ATM-dependent G2/M checkpoint system.

### Squalene is not a direct inhibitor of ATM and ATR kinase activities

**Fig 5:**
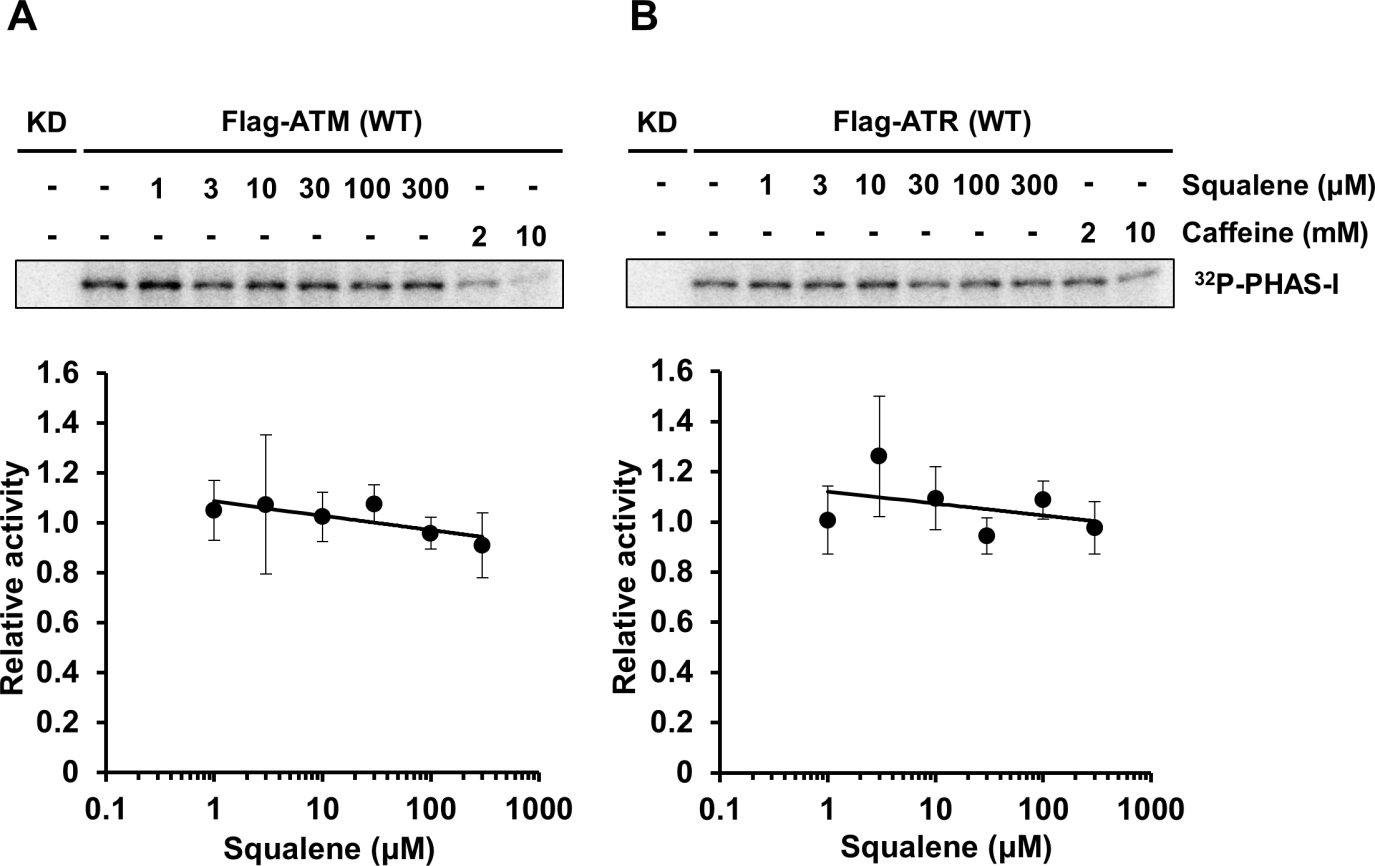
Squalene does not inhibit ATM and ATR kinases. ATM (A) and ATR (B) kinase activities were measured *in vitro* using PHAS-I as the substrate in the presence of squalene (1, 3, 10, 30, 100, and 300 μM). HEK293T cells were transfected with flag-tagged ATM- or ATR-wt plasmid. ATM and ATR protein kinases were purified by immunoprecipitation using anti-Flag^®^ M2 antibody and protein-G sepharose. Kinase activity was monitored for 20 min at 30°C. Data are presented as the mean ± S.D. of three independent experiments. Caffeine (2 and 10 mM) was used as an ATM/ATR inhibitor.

To address whether or not squalene directly inhibits ATM and ATR protein kinases, its inhibitory activity was studied by *in vitro* kinase assay using immunoprecipitated ATM and ATR derived from HEK293T cells. ATM and ATR kinase activities were measured using PHAS-I as a specific substrate for the endogenous Flag-ATM and Flag-ATR purified by immunoprecipitation. Caffeine, a ATM/ATR inhibitor, inhibited ATM and ATR kinase activity. However, we found that squalene did not inhibit the kinase activity of ATM and ATR (Fig 5A and 5B).

### Squalene increases the intracellular Wip1 protein level

**Fig 6:**
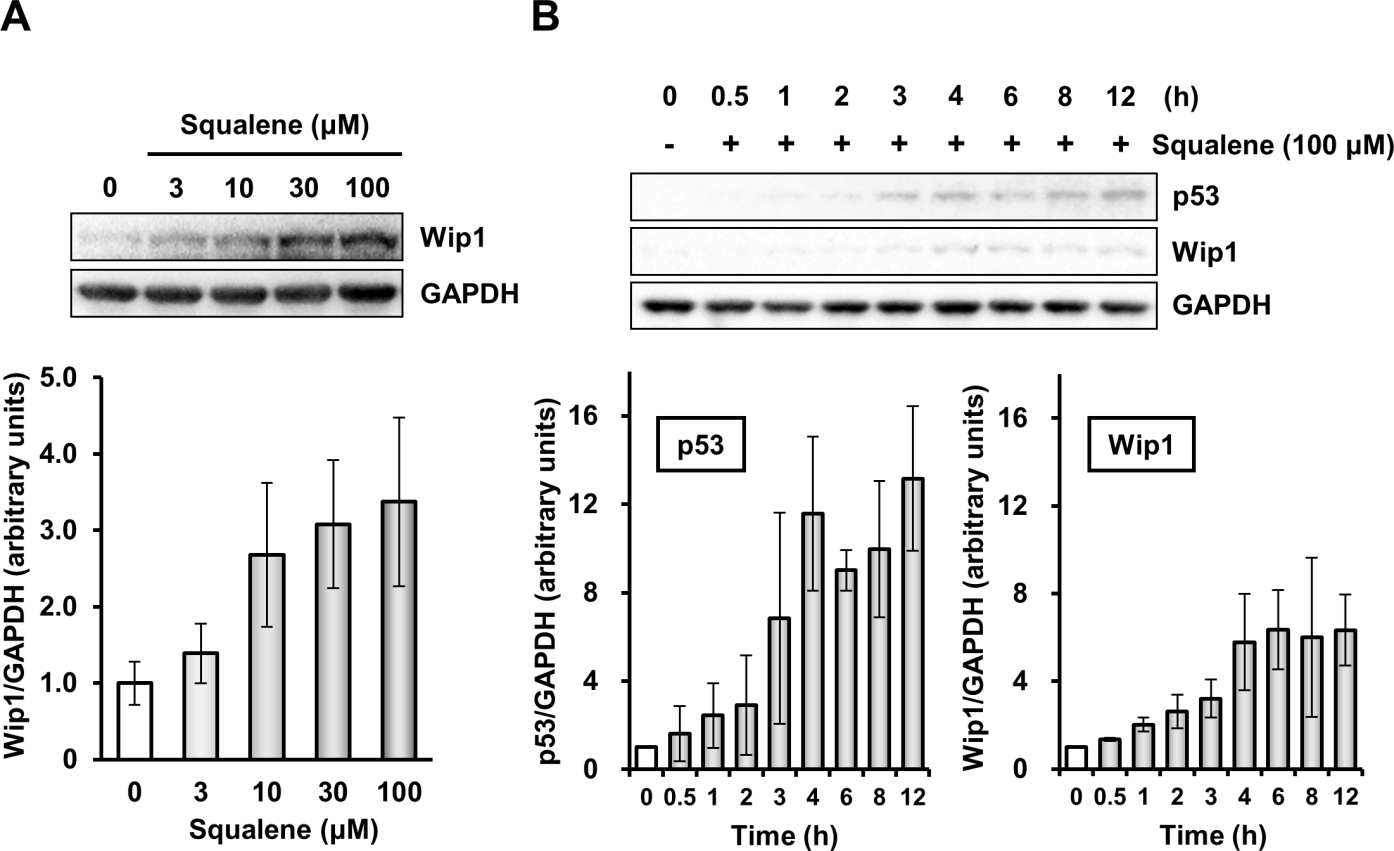
Squalene increases Wip1 expression in a concentration- and time-dependent manner. (A) A549 adenocarcinoma cells were incubated with squalene (0, 3, 10, 30, and 100 μM) for 4 h. (B) A549 adenocarcinoma cells were incubated with squalene (100 μM) for 0 to 12 h. Expression of p53 and Wip1 expression was examined by immunoblot analysis. GAPDH was used as a loading control. Densitometric analysis of the bands was performed using Image Quant 5.2 software.

Because *in vitro* ATM kinase activity was not directly inhibited by squalene, an indirect mechanism might be involved in the regulation of ATM activity, and thus we focused our attention on Wip1 phosphatase, which negatively regulates ATM activity via dephosphorylation. Squalene induced upregulation of Wip1 phosphatase in A549 cells in a concentration-dependent manner (Fig 6A). It has been reported that Wip1 is induced by γIR in a p53-dependent manner [28] and that Wip1 expression is associated with the stabilization of p53. In the present study, expression of both p53 and Wip1 increased up to 4 h after squalene treatment in A549 cells and was maintained for 12 h (Fig 6B). These results indicate that squalene was involved in Wip1 protein induction at the transcriptional or post-transcriptional levels.

## Discussion

DNA damage checkpoints are self-monitoring systems that prevent the carry-over of damaged DNA to the next generation of cells. When DNA damage occurs, these checkpoints are activated either to arrest the cell cycle or to lead the cells into apoptosis [1,2]. The necessity of ATM for the activation of DNA damage checkpoints and for the survival of cells following exposure to IR, UVC radiation, and anticancer drugs is well recorded [29–31]. Because disruption of DNA-damage checkpoints can result in enhanced chemo- and radio-sensitization of cancer cells [8,32], the discovery and development of inhibitors of checkpoint kinases, in particular, the proximal ATM kinase, should prove beneficial as a radio/chemo-sensitizing therapy for effective killing of cancer cells [8,11,33].

In the present study, we demonstrated that squalene significantly enhanced cellular sensitivity to UVC and γIR (Fig 2B and 2C), and the effect was concentration-dependent (Fig 2C), suggesting that squalene influenced the radiation-induced DNA damage response. It is known that two master controller kinases of the DNA damage checkpoint signaling pathway, ATM and ATR, respond to different types of DNA damage [1,2]. ATR is mainly activated by single-strand breaks (SSBs) produced by UVC or DNA damaging agents, whereas ATM responds to DSBs produced by IR [2,32] and high doses of UVC [34]. A higher UV dose may trigger ATM activation [35,36] (Fig 2B). Consistent with the obvious decrease in cell survival induced by squalene following IR, squalene treatment disrupted the G2/M checkpoint signal activated by IR exposure, thereby suggesting that squalene might have an inhibitory effect on the ATM-dependent checkpoint rather than on the ATR-dependent checkpoint. It has been reported that ATM activation occurs within minutes following IR exposure [37]. Because mitotic cells were detected 1 h after γIR in our G2/M checkpoint study (Fig 4), the inhibitory effect of squalene was attributed to the participation of ATM induced by γIR. Taken together, these results unequivocally support the ATM kinase-specific inhibitory effect of squalene in the DNA damage response.

To determine whether squalene acts directly on kinase activity, we measured *in vitro* ATM and ATR kinase activity in the presence of squalene. Neither kinase was a specific target of squalene (Fig 5). It was thus suggested that squalene indirectly modulates ATM activation after DNA damage.

It has been reported that inactivation of ATM is strictly regulated by Wip1 phosphatase, which de-phosphorylates the activation motif of ATM [7]. Indeed, the present study revealed that squalene treatment of A549 cells significantly increased Wip1 protein expression in a time-dependent manner (Fig 6). Fiscella *et al*. reported that expression of Wip1 phosphatase is stimulated by the stabilization of p53 [28]. Here, we also found that Wip1 expression was associated with the intracellular stabilization of p53. Importantly, signaling of ATM and its downstream checkpoint molecules occurs through specific phosphorylation sites following DNA damage. Although squalene concentration-dependently increased Wip1 expression or stabilized Wip1 protein, γIR-induced phosphorylation of p53, Chk1, Chk2, and SMC1 was clearly reduced by squalene treatment. Wip1 is known to dephosphorylate the SQ/TQ motifs of Chk2 (Thr68), Chk1 (Ser317, Ser345), and p53 (Ser15) [38–40]. Therefore, squalene has the potential to inhibit two main pathways. We demonstrated that squalene inhibits the ATM-Chk2-p53 pathway (Fig 3B). However, phosphorylation of ATR was not clearly observed in γIR-irradiated cells (Fig 3C). Our results suggest that squalene preferentially inhibits ATM activity when γIR-induced DNA damage occurs, but may also be involved in ATR inhibition in the response to other types of DNA damage such as single-strand DNA breaks. Further studies are necessary to characterize these effects. Additionally, squalene itself did not affect the cell cycle distribution in unperturbed A549 cells (Figs 3 and 4). Thus, we suggest that squalene-induced Wip1 expression contributes to the regulation of γIR-induced ATM-dependent signaling during the DNA damage response.

In recent years, although vigorous efforts have been made to develop DNA damage checkpoint inhibitors that can be used as sensitizers for anticancer therapy, most drug candidates are not suitable for clinical application because of their lack of specificity [11]. Approximately 74% of anticancer drugs used clinically from 1981 to 2002 were compounds derived from natural products or mimetics [41]. Squalene is a biochemical component that is synthesized [42] and distributed in various human organs, such as the liver, lung, pancreas, kidney, and skin [43]; thus, no side effects would be expected with squalene, in contrast to synthetic drugs. Although the pharmacological profile of Squalene has yet to be fully defined, we found that squalene treatment at concentrations above 100 μM did not decrease cell proliferation (Fig 2A).

As such, squalene may be a desirable sensitizer for cancer therapy because it is an endogenous molecule that inhibits ATM with minimal side effects. In fact, high doses of squalene resulted in 95% cell viability in our present study; therefore, squalene is an indirect inhibitor of DNA damage during cancer therapy without side effects. Squalene might therefore also be used for sensitizing malignant cells to anticancer drugs associated with ATM activation, e.g., DNA-methylating agents [44], DNA-alkylating agents [45,46], and topoisomerase inhibitors [47,48]. Lastly, by inhibiting the G2/M checkpoint, squalene could be used along with anticancer therapies to specifically target cancer cells that are resistant to chemotherapy and radiotherapy as a result of the absence of functional p53 [49–51]. Further investigations are needed to determine the feasibility of the clinical use of squalene as a sensitizing agent in anticancer therapies.
